# Diff-seq: A high throughput sequencing-based mismatch detection assay for DNA variant enrichment and discovery

**DOI:** 10.1093/nar/gky022

**Published:** 2018-01-19

**Authors:** Dimitra Aggeli, Vlad O Karas, Nicholas A Sinnott-Armstrong, Vici Varghese, Robert W Shafer, William J Greenleaf, Gavin Sherlock

**Affiliations:** 1 Department of Genetics, Stanford University School of Medicine, Stanford, CA 94305, USA; 2 Department of Medicine, Stanford University School of Medicine, Stanford, CA 94305, USA; 3 Department of Applied Physics, Stanford University, Stanford, CA 94305, USA; 4 Chan Zuckerberg Biohub, San Francisco, CA, USA

## Abstract

Much of the within species genetic variation is in the form of single nucleotide polymorphisms (SNPs), typically detected by whole genome sequencing (WGS) or microarray-based technologies. However, WGS produces mostly uninformative reads that perfectly match the reference, while microarrays require genome-specific reagents. We have developed Diff-seq, a sequencing-based mismatch detection assay for SNP discovery without the requirement for specialized nucleic-acid reagents. Diff-seq leverages the Surveyor endonuclease to cleave mismatched DNA molecules that are generated after cross-annealing of a complex pool of DNA fragments. Sequencing libraries enriched for Surveyor-cleaved molecules result in increased coverage at the variant sites. Diff-seq detected all mismatches present in an initial test substrate, with specific enrichment dependent on the identity and context of the variation. Application to viral sequences resulted in increased observation of variant alleles in a biologically relevant context. Diff-Seq has the potential to increase the sensitivity and efficiency of high-throughput sequencing in the detection of variation.

## INTRODUCTION

The rapid advances in low-cost, high-throughput sequencing have enabled numerous resequencing applications, ranging from clinical oncology (1) to evolutionary dynamics (2,3). For many such applications, the goal of resequencing is the identification of sequence variants in a population of different genomes. This polymorphism detection problem often requires brute-force, high-depth shotgun sequencing of genomic DNA isolated from a population of cells, and painstaking bioinformatics analyses to confidently identify real genetic polymorphisms from a background of sequencing errors. For rare or infrequent polymorphisms, this approach often results in an overwhelming excess of reads that exactly match the reference genome, whereas reads containing true variants are only a tiny fraction of the total (4–6). Even for small genomes, such as viral genomes, several hundred-fold coverage is required for confident detection of variants present at ∼1% frequency (7,8), while techniques that enable variant calling well below the error rate of the platform require extremely high coverage data (9) or engineered redundancies in sequencing (often involving molecular barcodes).

If the specific polymorphism to be detected is known *a priori*, a variety of powerful and elegant approaches designed to detect specific locations of sequence variation may be employed. For example, rolling circle amplification (10), molecular inversion probes (11) and mismatch ligation with bioluminescence detection (12), all based on DNA ligase and often on nuclease activities, can be used to detect the presence of specific alleles. Similarly, Taqman, molecular beacons, and related assays can also be used to detect specific, targeted alleles (13–17). However, all of these methods require *a priori* knowledge of the reference sequence of the genome and/or alleles under interrogation, and often involve the construction of sophisticated probes to detect individual alleles.

By contrast, mismatch detection assays rely on the base-pairing quality of DNA, and subsequent enzymatic detection of mispaired bases (18–21), and are thus agnostic to the exact identity of the underlying mutation. Mismatch endonucleases act on the mismatched sites of heterohybrid DNA, generated by denaturation and reannealing of a population of DNA molecules, to produce fragments resolvable by electrophoresis, enabling detection of variation across whole genes, or even across small genomes (22–24). Genomic mismatch scanning and other platforms, such as the tiling array and the mismatch endonuclease array-based methodology (MENA) use DNA hybridization and mismatch endonucleases to uncover single nucleotide polymorphisms (SNPs) at genomic scales (25–29).

We aimed to couple mismatch detection with high-throughput sequencing to allow for the detection of polymorphisms across a DNA sample. This *de novo* polymorphism detection method allows for the identification of variation that could occur anywhere in a genome, and furthermore specifically targets sequencing capacity to the variant positions and their genomic context. Our method, which we refer to as differential sequencing (Diff-seq), aims to increase the sensitivity of high throughput sequencing for the detection of rare variation, and can be directly applied to small genomes or amplicons.

The enzymatic foundation of Diff-seq is the Surveyor endonuclease, which cuts heterohybrid DNA molecules at the sites of mispaired bases. By denaturing and reannealing a complex pool of DNA fragments, we generate a pool of heterohybrid double stranded DNA (dsDNA) molecules, which contain mismatches at positions of genetic variation. These heterohybrids are then digested with Surveyor endonuclease, and the generated fragments are targeted for inclusion in a high-throughput sequencing library, resulting in substantial enrichment for DNA fragments with polymorphic sites. Diff-seq thus enables the identification of the variant position within the sequencing read, and determination of the variant base.

We first applied Diff-seq to a simple 1 kb test substrate with 0–4 mismatches to demonstrate its efficacy, then further demonstrated its performance on simple but mutation-dense populations of Human Immunodeficiency Virus (HIV) molecules. Diff-seq enabled the detection of polymorphic sites between two clones when the clones were mixed in a variety of stoichiometries. We finally applied Diff-seq to DNA molecules derived from HIV population samples (8), and showed that Diff-seq can increase the observation frequency of variant positions in biologically relevant samples.

## MATERIALS AND METHODS

### Preparation and amplification of 1 kb model substrate

pET17b (Novagen, Madison, WI, USA) derivatives were generated by the introduction of single point mutations via QuickChange PCR (primers in Supplementary Table S1) and cloning into *E. coli*. 1 kb variants were amplified in 50 μl reactions from 4 ng of either pET17b or derivatives, using PrimeSTAR (TaKaRa, Mountain View, CA, USA) and primers VK41 and VK42 (Supplementary Table S1), each at a final concentration of 0.4 μM, in the following conditions: 98°C for 10′, 35 cycles of 98°C for 10”, 55°C for 5”, 72°C for 1′.

### Preparation of DNA from viral clones and populations

Viral clones, whose sequences included the reverse transcriptase, integrase and protease regions of the *pol* gene, were a generous gift of Mark Winters and Mark Holodniy. The clones were amplified in 50 μl reactions from 4 ng of each clone with PrimeSTAR and primers DAo43 and DAo44 (Supplementary Table S1), each at a final concentration of 0.4 μM, in the following conditions: 98°C for 10′, 30 cycles of 98°C for 10”, 59.5°C for 5”, 72°C for 2′.

RNA preparation, RT-PCR and amplification of the population viral genomes have been described previously (8), with the exception that a single ∼1 kb amplicon, including the reverse transcriptase and protease-encoding sequences, was generated in a single PCR. The consensus sequence of the population with PID 5248 (8) was synthesized by Life Technologies (Carlsbad CA) and had the most frequently appearing nucleotide at every position, as determined by Sanger sequencing.

### Diff-seq

**Figure 1.**
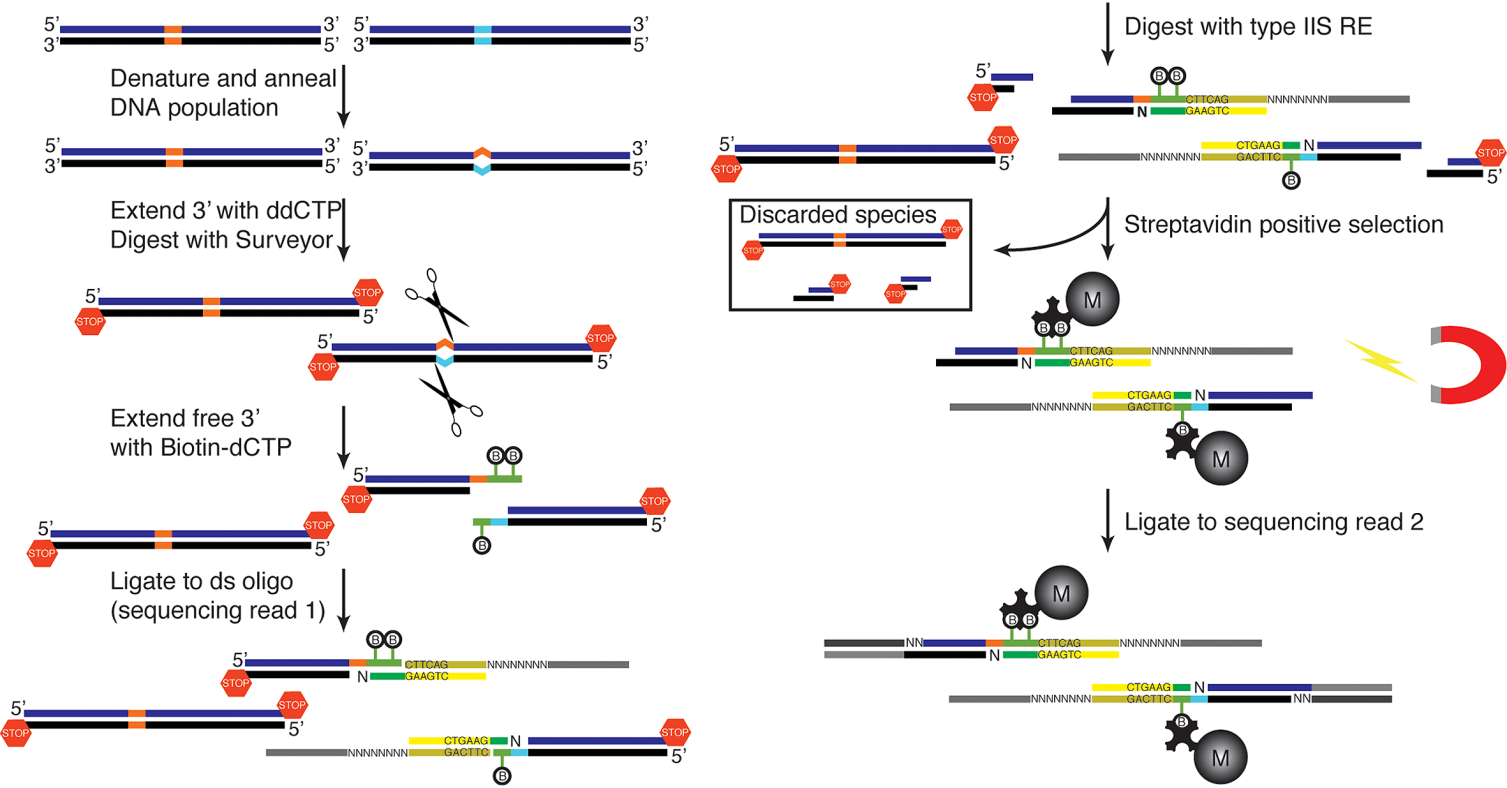
Differential sequencing method. Hybrid DNA molecules are generated by thermal melting and reannealing. Mismatched molecules become substrate for Surveyor and are tagged with a biotinylated nucleotide for subsequent selection. Sequencing adaptors are introduced in two ligation steps. A type IIS recognition site, engineered in the first ligating oligo, is used to ensure a homogeneously sized library. After the second ligation step the library is amplified, quantified with qPCR and sequenced.

The overall protocol, whose steps are described below, is summarized in Figure 1.

All purification steps were carried out using the MinElute Reaction Cleanup Kit (QIAGEN, Redwood City, CA, USA) and the products were eluted in 20 μl EB buffer, unless otherwise stated. Oligonucleotides were synthesized by IDT. Double stranded oligonucleotides used in the ligation reactions were generated by mixing complementary single stranded oligonucleotides isostoichiometrically in 50 mM NaCl and 10 mM Tris pH 7.5, boiling at 95°C for 5′ and incubating at room temperature for 30′.

#### Reannealing reaction

PCR products were purified with QIAquick PCR Purification Kit (QIAGEN, Redwood City, CA, USA), concentrated to 150–200 ng/μl and the dsDNA concentrations were estimated with a Qubit fluorometer using the Quant-iT dsDNA HS kit (Invitrogen, Waltham, MA, USA). A total of 3 μg of DNA was denatured at 95°C for 10′ and reannealed by decreasing the temperature by 5°C every 10′ down to 20°C, on a thermocycler, in 10 mM Tris pH 7.5 and 50 mM NaCl and at a DNA concentration of 125–150 ng/μl.

#### S1 nuclease digestion

The reannealed products were digested with 50 units of S1 Nuclease (Thermo Fisher Scientific, Santa Clara, CA, USA) per μg of DNA in 1X S1 buffer at 40–60 ng/μl DNA concentration for 1 h 30′ at 25°C. The digested products were purified with the QIAquick PCR purification kit and eluted in 50 μl EB buffer.

#### Blocking 3′ ends with 2’,3’-dideoxycytidine-5’-triphosphate (ddCTP) and Terminal Transferase (TdT)

The 3′ ends were blocked with 3–5 nmol ddCTP (Affymetrix, Santa Clara, CA, USA) and 20 units TdT (NEB, Ipswich, MA, USA) in 64 μl 1× TdT buffer supplemented with 0.25 mM CoCl_2_, for 1 h at 37°C, and the products were purified.

#### Mismatch digestion

800–1000 ng DNA, which is approximately half the amount that was recovered from the previous reaction, were digested with 2 μl Surveyor endonuclease, in the presence of 2 μl Enhancer (Mutation Detection Kit, IDT, Redwood City, CA, USA), and 20 units Ampligase (Epicentre, Madison, WI, USA) in 1X Ampligase buffer and DNA concentration of 20–25 ng/μl for 50 min at 42°C, and the products were purified.

#### 3′ end extension with biotin-14-dCTPnucleotides (B-dCTP) and TdT

The newly generated 3′ ends were extended with 80–140 pmol B-14-dCTP (Invitrogen, Waltham, MA, USA) and 20 units TdT in 27 μl 1× TdT buffer supplemented with 0.25 mM CoCl_2_ for 1 h at 37°C, and the products were purified.

#### Ligation

Each of the oligos DAo83, DAo84 and DAo85 was reannealed to oligo DAo97 and the resulting double stranded oligos were mixed isostoichiometrically to yield the ligating oligo pool (6.7 μM per species). The purified DNA products were ligated to 1.5 μl ligating oligo pool in the presence of 2000 units T4 ligase (NEB, Ipswich, MA, USA) in 33 μl 1× T4 ligase buffer and 10% polyethylene glycol (PEG) 4000 for a minimum of 5 h at 16°C, and the products were purified.

#### Type IIS restriction endonuclease digestion

The DNA was digested with 50 units AcuI endonuclease (NEB, Ipswich MA) in 25 μl 1× CutSmart buffer supplemented with 65 μM *S*-adenosylmethionine at 37°C for a minimum of 4 h. AcuI recognition sites were introduced during the previous step and digestion resulted in a homogeneously-sized library.

#### Biotinylated fragments pulldown with streptavidin magnetic beads

25 μl M270 beads (Invitrogen, Waltham, MA, USA), were washed twice in Bind and Wash buffer (B&W, 5 mM Tris pH 7.5, 1 M NaCl, 0.5 mM EDTA) and resuspended in 25 μl 2× B&W. The beads were then added to the AcuI reaction and the slurry was incubated for 1 h 30′ in a roller drum at room temperature. The beads were washed once in 1× B&W + 0.1% Tween 20, three times in 1× B&W, once in 1× saline-sodium citrate (SSC) buffer (Sigma-Aldrich, St Louis, MO, USA) and once in water.

#### Ligation

The washed beads were resuspended in 10 μl ligation reaction (2000 units T4 ligase (NEB, Ipswich, MA, USA), ∼50 pmol ds oligo DAo98/DAo99 in 1× ligation buffer and 10% PEG 4000). The reactions were incubated at 16°C for a minimum of 5 h on a rocker. The beads were then washed once in 1× B&W + 0.1% Tween 20, three times in 1× B&W, once in 1× SSC buffer (Sigma-Aldrich, St Louis, MO, USA) and resuspended in 20 μl water.

#### Amplification and library sequencing

5 μl of the bead suspension was used in an amplification reaction to introduce Nextera Illumina sequencing adaptors and read indices to the biotinylated DNA strand for multiplexed sequencing (Supplementary Table S1). The fragments were amplified for 6 cycles with PrimeSTAR in a 50 μl reaction, using the following PCR conditions: 98°C for 10′, 6 cycles of 98°C for 10”, 66°C for 5”, 72°C for 10". The amplification product was purified and quantified in a One Step qPCR instrument (Thermo Fisher Scientific, Santa Clara, CA, USA) against PhiX Control v3 (Illumina, Santa Clara, CA, USA), with Illumina flow cell adaptor sequences (Supplementary Table S1) to determine the number of amplification cycles available prior to PCR saturation. The cycling conditions were 98°C for 10′, 30 cycles of 98°C for 10”, 63°C for 5”, 72°C for 10". A fraction of the rest of the library was re-amplified using conditions identical to the qPCR conditions for another 10–12 cycles, then purified, quantified once more by qPCR against PhiX and paired-end sequenced on a MiSeq instrument for 150 cycles.

### Processing of sequencing reads

Processing of sequencing reads prior to downstream analysis is depicted in Supplementary Figure S1. Briefly, paired-end reads were merged using FLASH version 1.2.11 (30). The merged reads were then deduplicated and adaptors and auxiliary sequences (introduced by the first ligation reaction, including the *AcuI* restriction site and the UMIs) were trimmed using cutadapt version 1.9.1 (31). Reads that were less than eight nucleotides long, and reads that did not start with a G were excluded from further analysis. The 5′ G was trimmed from the remaining reads, which were then aligned to the reference sequence using bowtie2 version 2.2.6 (32).

### Nextera libraries preparation

Nextera libraries were prepared using a modified Illumina Nextera protocol as described (33) and paired-end sequenced on a MiSeq for 150 cycles. The data were analyzed and processed using a custom python script in conjunction with other freely available software. Briefly, the sequencing reads were trimmed using cutadapt version 1.9.1 (31), then quality- and length-filtered. The filtered reads were then aligned to the reference sequence with bwa version 0.7.15 (34), and the aligned reads were sorted and indexed using Picard version 2.7.1 (http://broadinstitute.github.io/picard). SNPs were called using the GATK software (35).

The values used in Figure 4 and Supplementary Figure S6 represent averages from 2 technical replicates for the Nextera data and for the Diff-seq data for dilution 1:1. The values representing the rest of the dilutions for Diff-seq data were each derived from a single experiment.

## RESULTS

### Method description

Diff-seq generates a sequencing library that is enriched for loci that are polymorphic in the input DNA (Figure 1, Supplementary Figure S1). To achieve this enrichment, Diff-seq harnesses the mismatch cleavage activity of the CELII endonuclease (commercially available as Surveyor Nuclease) (23). First, a population of DNA molecules is thermally denatured and then reannealed by gradual return to 20°C, to create mismatch-containing heterohybrid molecules. S1 nuclease is used to eliminate poorly reannealed molecules and excess single-stranded DNA. Free 3′ ends are extended with ddCTP using TdT, which blocks them from participating in subsequent enzymatic steps. The reannealed DNA is then digested using Surveyor, which specifically cuts both strands 3′ of a mismatch position (23,24), resulting in dsDNA molecules with a reactive 3′ overhang at one end that should correspond to the mismatched base(s). Surveyor digestion is carried out in the presence of Ampligase to reduce non-specific Surveyor cleavage (36). The newly-generated 3′ ends are then extended with B-dCTP using TdT. These extended 3′ ends are then used as substrates for a ligation reaction that introduces: (i) the primer sequence for the forward Illumina sequencing read (along with some buffer sequence after the B-dCTP extension), (ii) unique molecular identifiers (UMIs) between the primer sequence and the buffer sequence and (iii) an AcuI (type IIS endonuclease) recognition site, oriented such that the cut site will be within the captured fragment. The ligation reaction is designed to capture fragments that had incorporated up to 3 B-dCTP nucleotides. AcuI restriction digestion then generates a homogeneously-sized library, eliminating potential PCR and sequencing size biases. After digestion, biotinylated fragments are captured using streptavidin magnetic beads. Then, while the library is still attached to the beads, a second ligation reaction introduces sequence for the reverse Illumina sequencing read. Finally, sequencing adaptors and library-specific indices are introduced via amplification directly from the bead-attached material. A small aliquot of this first round of amplification is used to monitor PCR saturation via qPCR with SybrGreen against the Illumina PhiX library, and determine the number of additional cycles remaining prior to saturation. After the second amplification, the final libraries are quantified by qPCR and then sequenced on an Illumina MiSeq.

The assay generates sequencing reads that align at and around variant positions (Supplementary Figures S2A and B). Supplementary Figure S2B shows the total coverage frequencies for a single mismatch case, and the fully-matched control. From the library structure (see Figure 1 and Supplementary Figure S1), we expected that the variant position should lie at the beginning of each processed, unaligned read, so we focused our efforts on calling variants on specific parts of the aligned read, rather than the whole read. We therefore converted the total coverage data to ‘Diff-seq coverage’, which we used for subsequent analyses. The first ligation step is designed to capture molecules extended with up to three biotinylated CTPs. However, even after G-trimming of the unaligned reads (see Materials and Methods), there is still uncertainty as to where within the next two or three bases the variant position lies when the reads start with G or GG, respectively, as the particular Gs may have been part of the extension, or may represent a variant base. Thus, we retained up to three consecutive bases (depending on their identity), located at the beginning of forward-aligned or at the end of reverse-aligned reads to generate the Diff-seq coverage track (Supplementary Figure S2C). Depending on the length of the extension, the context of the mismatch, as well as the identity of the variation itself, signal on neighboring positions can be comparable to the signal in the variant position, as shown in the next section.

The efficiency of the Diff-seq protocol, calculated as the number of unique molecules that mapped to the reference genome, divided by the calculated number of molecules present at the beginning of the experiment, was ∼0.00065% using 3 μg of starting material (Supplementary Table S2). We assumed that only half of the molecules made it into the library preparation (only the heterohybrids), and that each of these molecules could result in exactly two sequencing reads (each one capturing the mismatch site from different orientations). This second assumption holds true only for molecules with a single polymorphic site.

### Application of Diff-seq to a simple genome

**Figure 2.**
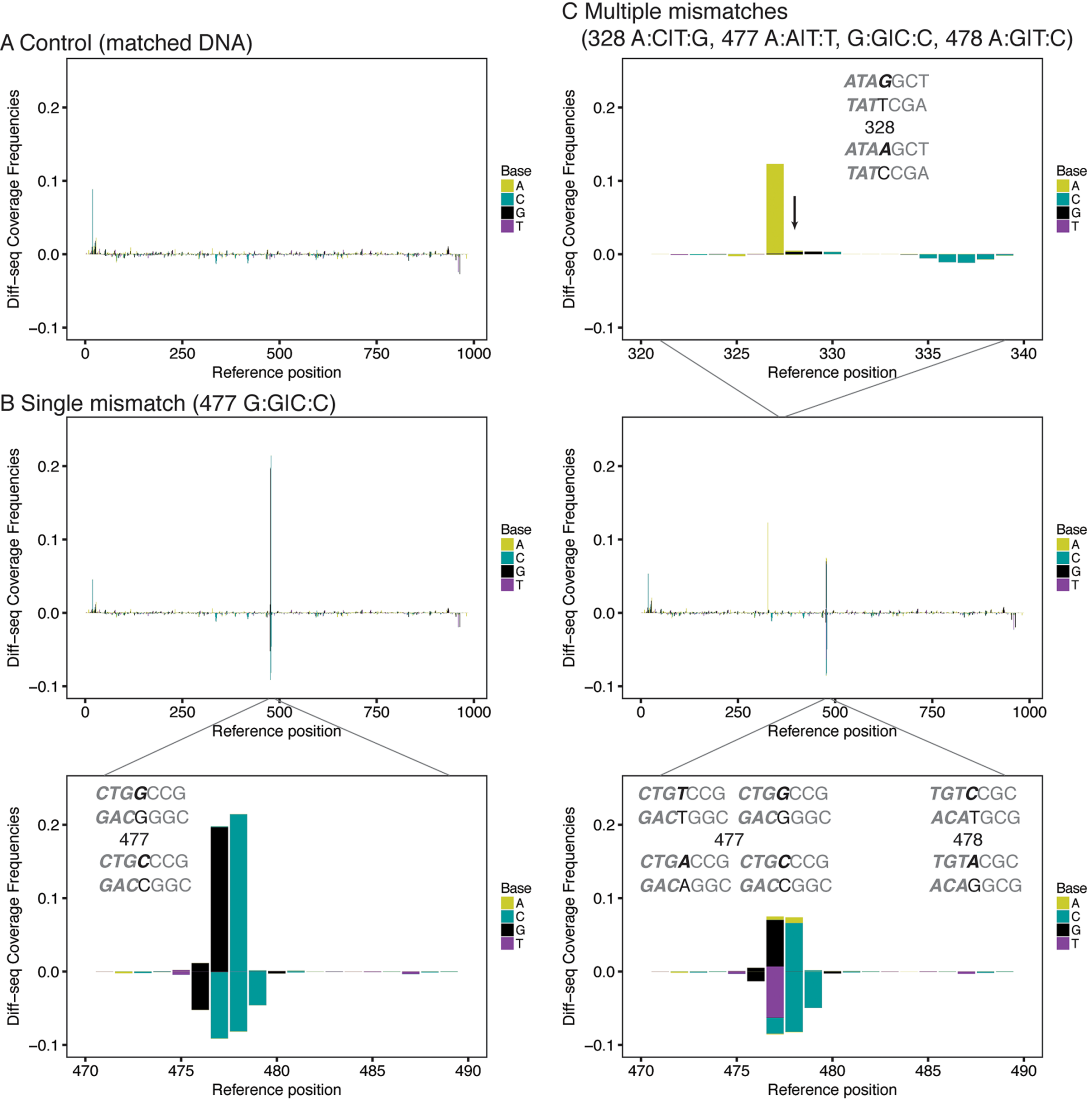
Differential sequencing application to a model substrate. Differential sequencing libraries derived from a 1 kb sequence were prepared as described in Figure 1 and sequenced. The Diff-seq coverage frequency for each position of the reference strand is plotted against the position, and color-coded according to the nucleotide base identity. Positive and negative values represent values for the forward and reverse strand, respectively. (**A**) Sample with no variation. (**B**) Sample with G:G|C:C variation at position 477. (**C**) Sample with multiple variant positions, assembled from four individually reannealed samples mixed isostoichiometrically. The arrow points to the variant position 328. For B and C, zoom-in the x-axis plots at the variant positions is shown. The context of the variation is annotated in gray in each graph with the mismatched bases in black and the strands that represent forward and reverse orientations are annotated with bold italicized and regular font, respectively.

To develop and test Diff-seq, we applied it to a known sequence with defined mismatches. This test substrate was a 985 bp sequence originating from the pET17b vector and derivatives containing single base-pair substitutions, which were introduced singly in three different positions. By assaying this simple substrate, we tested the extent to which the method can detect different types of variation, and examined biases introduced by the context of the variation (Figure 2 and Supplementary Figure S3). All Diff-seq libraries were compared to a library generated from DNA fragments that did not contain mismatches (Figure 2A, Supplementary Figure S3F). Variants covering all possible substitutions at a single position were used to test the extent to which Diff-seq could detect different pairs of variants (Figure 2B and Supplementary Figures S3A–C). Identical mismatch types were also introduced into 2 different positions to determine if the local sequence context affects Diff-seq signal (Supplementary Figures S3D and E). Finally, a more complex sample comprising a mixture of all possible mismatch types was also assayed to test the capacity for their simultaneous detection (Figure 2C). The libraries were prepared, sequenced and processed as described above and the resulting numbers of reads for each library at each step are summarized in Supplementary Table S2.

Variation introduced at position 477 of the pET17b fragment was used to examine identity-based biases; our data show that G/C variation generally gives more signal than other variant combinations (Figure 2B and Supplementary Figures S3A–C), consistent with a Surveyor nuclease preference for G:G/C:C mismatched pairs (23,24).

Earlier work has suggested context-dependent Surveyor digestion activity of mismatched DNA (24). To examine our method for possible biases due to sequence context surrounding the variant position, we assayed samples that contained a single type of variation within two different contexts (positions 328 and 477 in Supplementary Figures S3D and E) on the same substrate. In order to ensure that each DNA molecule had up to one mismatch only, the samples were generated by isostoichiometrically mixing the relevant populations post-reannealing. Surprisingly, we found that variation at position 328 resulted in Diff-seq coverage predominantly at position 327, regardless of the exact identity of the contributing bases at position 328, suggesting that Diff-seq signal is context-dependent. We attribute this to possible context-dependent hotspots for detection by the method and/or favorable DNA reannealing alternatives.

Finally, we assayed all four variant types (alleles G and C at position 477, alleles A and T also at position 477, alleles C and A at position 478 and alleles G and A at position 328) by isostoichiometrically mixing appropriate combinations of reannealed molecules. We observed contributions from each relevant base from all variant pairs, suggesting that Diff-seq can identify multiple variant types in a complex mixture of molecules (Figure 2C).

### Diff-seq application to viral sequences

**Figure 3.**
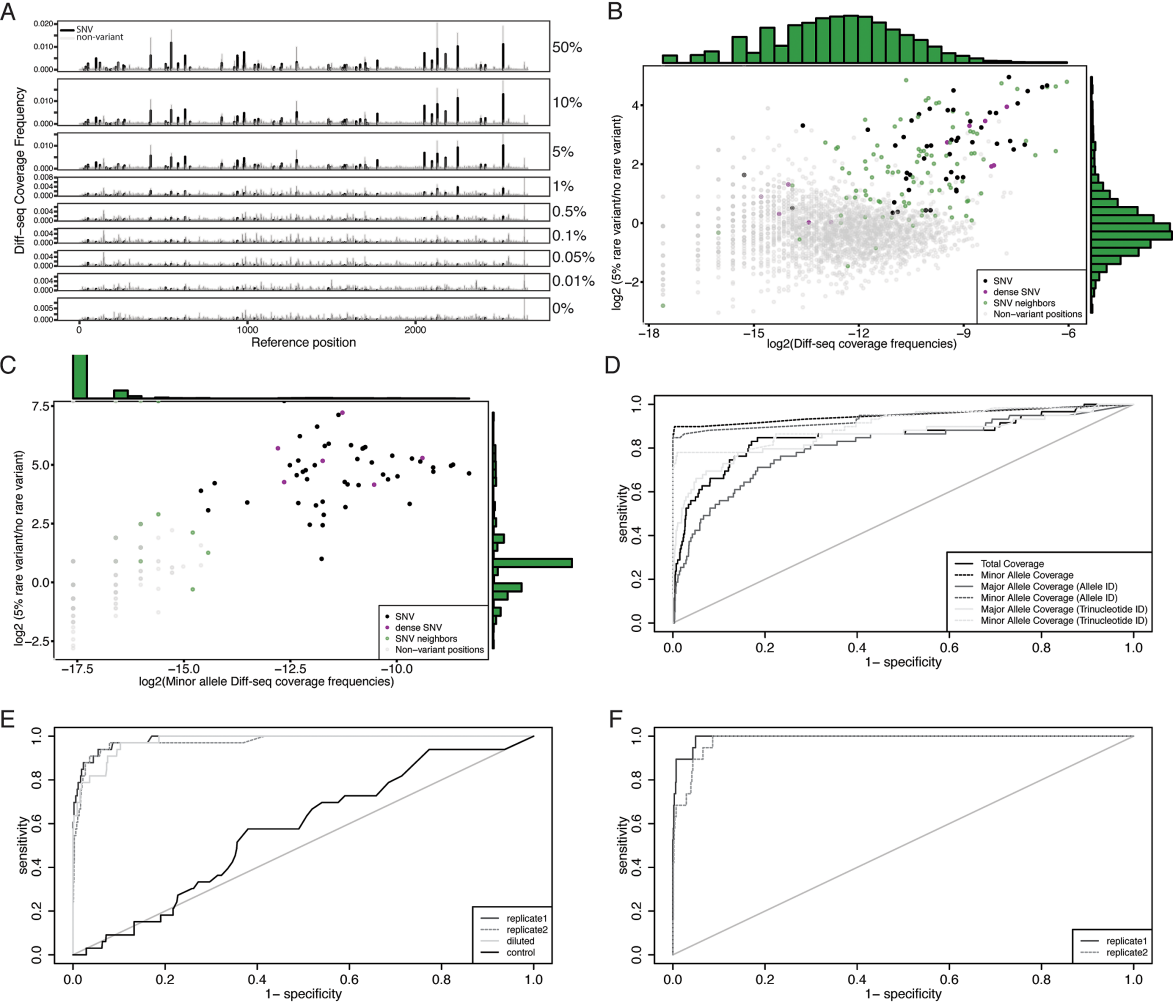
Differential sequencing application to viral genomes. (**A**) Differential sequencing libraries derived from a mixture of 2 viral clones were prepared as described in Figure 1 and sequenced on an Illumina MiSeq. The Diff-seq coverage frequencies, aggregated across both strands, are shown for a series of experiments where the contribution of the rare variant ranges from 50% to 0. Panels B–D focus on the 5% rare clone frequency dataset. (**B** and **C**) The log_2_ differential Diff-seq coverage between the 5% rare variant frequency and the control datasets were plotted against the log_2_ Diff-seq coverage per position for all positions of the template, considering (B) all and (C) the minor alleles. (**D**) ROC curves for the polymorphic sites called using different variables as predictors. The variables used are the sums of the per strand Diff-seq coverage enrichment scores per position (see text for details). (**E** and **F**) ROC curves for the sums of the per strand minor allele Diff-seq coverage enrichment scores per position, for PCR amplified viral population samples after Diff-seq application. (E) The sample corresponds to PID 5248 (8). The consensus sequence served as control and as a high-frequency variant, within which the population was diluted to 20%. (F) The sample corresponds to PID 30269 (8).

We next applied Diff-seq to viral genome-derived sequences, in order to determine our ability to identify multiple variants within a single DNA molecule, at a range of abundances (Figure 3, Supplementary Table S1). First, we applied Diff-seq to two HIV clones of size 2.7 kb, that originated from a single individual before and after treatment with antivirals (Figure 3A–D). The two clones differ at 59 positions, all of which are SNPs (with 11 located within 2 nucleotides of other SNPs). One clone was mixed with the other at varying relative abundances: 50%, 10%, 5%, 1%, 0.5%, 0.1%, 0.05%, 0.01% and 0%. We observed substantial signal when the minority clone is present at as low as 5% frequency, with substantial signal degradation upon further dilution, though some variants still give Diff-seq coverage at even lower frequencies (Figure 3A).

For the 5% minority variant frequency dataset, we derived mismatch- and allele-specific contributions to the total and strand-specific signals for each of the variant positions (Supplementary Figure S4A). The mismatch bias, shown in the first column, shows how much detection of the one mismatched pair is favored over the other. The forward and reverse allele biases, shown in the next two columns, represent strand-specific biases for the alternative allele. From these we derived the average and maximum allele bias as figures of merit for variation detection. We also calculated the total allele bias, generated from the total reads aligning to the alternative allele on either strand compared to all reads aligning to reference. The sites have been ordered first by the identity of contributing alleles and then by the trinucleotide context. These data suggest that the detection of one mismatch pair or allele is often favored over the other, and this preference is consistent across positions carrying the same variation. The last column shows the log_2_ Diff-seq coverage frequencies. This coverage is largely independent of the variation and its context. In particular, five out of the eight least covered SNP positions are located within a very mutation-dense region of the substrate (positions 183, 184, 187, 188 and 190).

We also estimated the contribution of the type of variation to the detection of each nucleotide, by calculating the average positional frequencies (across any trinucleotide context) of each relevant base for the 59 SNP positions of the 50% dataset, binned by type (Supplementary Figure S4B). These data suggest that the contribution of each nucleotide to the total signal is independent of the variation type. In particular, a G nucleotide gives the majority of the signal regardless of the identity of the other allele, whereas, a mismatched C will give the least amount of signal, also regardless of the identity of the other allele (compare C reverse and G forward plots to C forward and G reverse plots).

To explore possible variant calling algorithms for our data, we plotted, for the 5% minority variant frequency dataset, the log_2_ ratio of Diff-seq coverage frequency for experiment vs. control (y-axis) against the log_2_ Diff-seq coverage frequencies in the experimental sample (x-axis) either for all alleles (Figure 3B) or just the minor alleles (Figure 3C). Most variant sites, along with variant neighboring sites are more highly covered on the 5% frequency dataset when all alleles are considered (Figure 3B). However, when only the minor allele is considered, there is a clear separation of the variant and non-variant sites (Figure 3C).

Based on these observations, we constructed potential SNP calling algorithms. We calculated z-scores for each position and strand, for the (i) total, (ii) minor allele, (iii) major allele depending on the nucleotide identity, (iv) minor allele depending on the nucleotide identity, (v) major allele depending on the trinucleotide context and (vi) minor allele depending on the trinucleotide context Diff-seq coverage. We used these per position z-scores (sum of z-scores of the forward and reverse strands) to generate ROC curves for the 5% frequency dataset (Figure 3D). The ranked *z*-scores for each of the variables used are shown in Supplementary Figure S5. We find that models that consider only the minor allele outperform those that consider the total coverage or the major allele coverage. Surprisingly, inclusion of allele identity and trinucleotide context (cases 4 and 6) did not increase the predictive power of the model.

To further test our SNP calling approach we applied Diff-seq to two samples, comprising populations of amplified HIV *pol* gene isolated from HIV patient plasma (8) (Figure 3E and F, see Supplementary Table S2 for sequencing library metrics). We also sequenced these populations with standard Nextera library preparation to identify variants in these samples. We employed the population consensus sequence for the sample with PID 5248 in Varghese *et al.* (8), both as a control and as ‘reference genome’ to dilute and reanneal against the population of molecules to be assayed (see Materials and Methods). The second population corresponds to the sample with PID 30269 in Varghese *et al.* (8). Our SNP calling method performed well for both population samples. It also performed comparably when the population sample with PID 5248 was diluted down to 20% frequency in the consensus sequence (Figure 3E).

### Comparison of Diff-seq to standard Nextera library preparation for variant discovery

**Figure 4.**
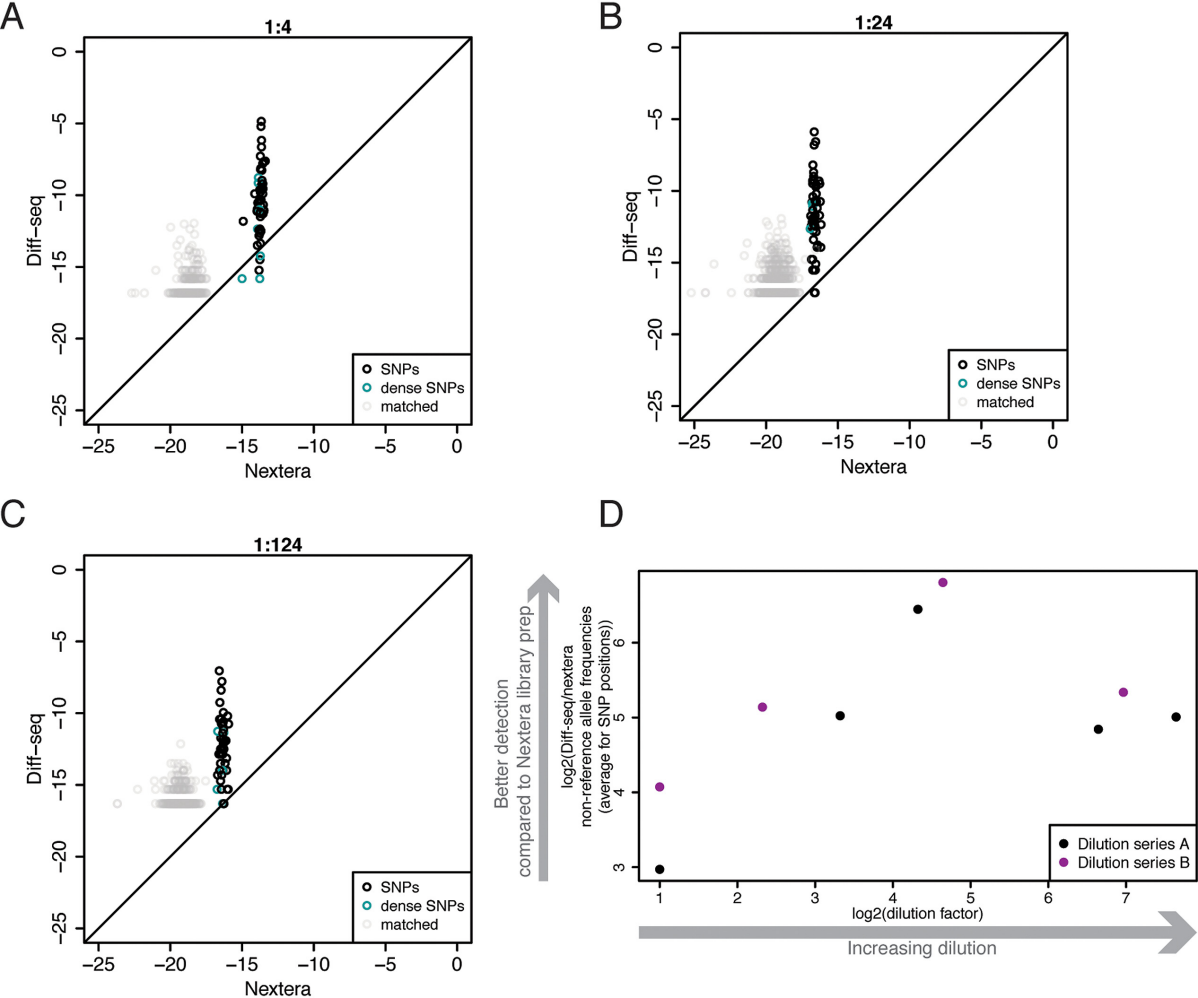
Comparison between Diff-seq and Nextera library preparations for the detection of variation in viral clones. (**A–C**) The log_2_ non-reference allele coverage frequencies for each position of the reference was plotted for the Diff-seq against Nextera library preparations. Non-variant positions, SNPs and dense SNPs are separately annotated. 1:4 (A), 1:24 (B) and 1:124 (C) rare:frequent molecule ratios are shown. (**D**) The log_2_ average of the ratios of the non-reference allele coverage frequencies Diff-seq/Nextera for the SNPs (positions shown in black or cyan in panels A–C, see Supplementary Figure S6 for all datasets) was plotted against the log_2_ dilution factor. Two Diff-seq sets of dilution series were employed and the points are colored accordingly.

To compare Diff-seq to standard, Nextera-based sequencing for variant discovery, we constructed Nextera libraries out of several dilutions of our clonal substrates (Diff-seq data shown in Figure 3A–D) and sequenced them on a MiSeq. We took into account the coverage as represented by the whole read for the Nextera data, and the Diff-seq coverage for the Diff-seq data (Supplementary Figure S2). Figure 4A–C shows the log_2_ frequencies of the non-reference alleles for all positions that were covered by both library preparations for non-reference:reference genome ratios 1:4, 1:24 and 1:124 (see Supplementary Figure S6 for a more complete set). The Nextera values for the non-variant positions, shown in grey, are presumably indicative of the MiSeq platform error rate. However, the corresponding Diff-seq values for the non-variant positions are increased. This detection of non-reference positions could either be the consequence of prior reactions, such as the extension reaction or due to misligation events, or result from true variant sites generated during amplification of the starting material. Our expectation was that the greater the dilution, the more the variant position cloud should deviate above the diagonal, as the Diff-seq data should be enriched in variant sites. For the 1:1 dilution (Supplementary Figure S6), although the SNP position cloud is located the furthest away from the error rate cloud, it does not deviate from the diagonal, and as expected the two methods perform similarly. Increasing the dilution factor, Diff-seq has an advantage in the detection of the rare variant (non-reference). The difference in the detection of rare variants between the two methods is summarized in Figure 4D, using the data shown in panels 4A–C and Supplementary Figure S6. Coverage frequencies of the rare variants can be 8–100× higher using Diff-seq compared to Nextera, depending on the dilution factor, when using only aligned reads and the Diff-seq coverage analysis. When the positional information of the Diff-seq dataset was not used and instead coverage derived from the whole read was considered, the rare variant frequencies were 4–14× higher for the Diff-seq data. Considering that the aligned reads were 25–60% of the total reads for the Diff-seq libraries, the number of reads ranged from being equal to and up to 8.5 times less than the Nextera library requirement.

We also compared the costs of the Diff-seq and Nextera library preparation. The cost per Nextera library has been estimated by Baym *et al.* (33) to be $8 per sample, excluding the sequencing reagents themselves. The cost for the Diff-seq per library was estimated to be $67 per sample, also excluding sequencing reagents, but including the two qPCR quality control runs and assuming that 10 samples were being processed simultaneously. However, the increased library costs are somewhat offset by the potential decreased costs associated with the sequencing reagents themselves. As noted in the previous paragraph, that decrease will depend on the frequency of the variants, and may be up to 8.5 times less than the Nextera library requirements.

## DISCUSSION

Here we describe Diff-seq, a new method for the detection of genetic variation within a population of DNA molecules. Diff-seq aims to outperform conventional high-throughput sequencing for SNP detection, though detection depends on both the identity of the contributing alleles and the context within which the variation arises. The method performed better with decreasing SNP density, while densely-spaced SNPs were not readily detected. The genomes we used did not contain indels, so the method was not tested for indel detection. It has been suggested that Surveyor endonuclease is less sensitive towards the detection of indels (37), so it would not be surprising if Diff-seq does not perform as well in that context.

One advantage of Diff-seq, compared to other approaches for polymorphism discovery, is that it is less sensitive to sequencing errors. Sequencing capacity in Diff-seq is targeted towards the mismatch endonuclease digestion sites, and polymorphic sites will appear in the first few nucleotides of the sequencing read, significantly limiting the sources of sequencing errors. Because of this fundamental advantage, we anticipate that further assay optimization will increase sensitivity of rare variation detection to extremely low frequencies.

We anticipate that polymorphism detection sensitivity may be improved through the use of an optimized reannealing protocol. Use of alternative endonucleases, such as T7E1 that perform better at indel positions (38), could expand the applicability of Diff-seq. Further optimization of other aspects of the method will likely also enhance its utility and ease of use. Currently the protocol requires eight enzymatic steps and a similar number of intermediate purification steps. Similarly to other methods, such as chromatin immunoprecipitation sequencing (ChIP-seq), each step is an opportunity for experimental variability and substrate loss. Decreasing or combining steps will certainly improve the efficiency of the protocol, and may also improve signal to background metrics. A stringent library size selection procedure prior to sequencing might also increase the fraction of usable reads and decrease the number of uninformative reads. Furthermore, the type IIS restriction enzyme currently used, AcuI, cuts only 16 bases away from its recognition site. For small genomes, such as the viral genomes used here, this small read size does not greatly affect mappability, however for larger genomes, unique mappability becomes problematic (39); a possible solution may be the use of combined sequence information from the forward and reverse reads that are adjacent to a specific polymorphism. MmeI and EcoP15I, which are type III restriction enzymes, have restriction sites further than 16 bases from their recognition site, yet also require two recognition sites in opposite orientation for efficient digestion, making them impractical for use in the Diff-seq protocol. To overcome this limitation, the type IIS restriction digestion might be eliminated altogether. However, in order to ligate the second sequencing adaptor, the end of the fragment would need to be rendered reactive, for example either by exonucleolytic cleavage of the dideoxynucleotide or by using a reversible terminator. Such an approach could, however, lead to the generation of highly variable DNA fragment lengths, which have strongly differential amplification and clustering efficiency on the Illumina flow cell. This variability might be partially alleviated by employing different sets of enzymes for restriction digestion for the generation of the initial fragments pre-reannealing.

As it currently stands, the Diff-seq library preparation is ∼8 times more expensive than the Nextera library preparation, because large amounts of multiple enzymes are needed, and there are multiple clean up steps. Finally, the quality control for Diff-seq is also a considerable part of the expense, with a current cost of $30 per qPCR run, which adds ∼$6 to the cost of a library. Future improvements should be targeted at increasing efficiency and decreasing these costs.

Diff-seq may be suitable for a variety of applications, from genotyping to estimating DNA polymerase error rates. In principle, any method applied to an already known genome can employ Diff-seq immediately prior to sequencing, to target sequencing capacity towards polymorphic sites, and in this way increase reads covering low frequency alleles. We note however that a potential limitation of the method is that it relies on the generation of mismatches for the detection of variation. Thus, Diff-seq using a diploid genome as the input DNA would only expected to detect heterozygous alleles, and would not identify homozygous or hemizygous variants. This limitation might be overcome by adding a known consensus sequence or reference genome to the sample.

Diff-seq application to more complex genomes will require robust DNA reannealing methods, such as oscillating phenol emulsion reassociation technique (osPERT) (40). Repetitive sequences reanneal more rapidly due to their higher relative abundance, and thus polymorphisms within these regions may be especially amenable to detection using Diff-seq. Alternatively, because of the kinetic separation of annealing times for repetitive regions, such regions can be removed (e.g. by retention on a hydroxyapatite column) (41). Additional annealing dynamics likely arise due to genome complexity, but how these will affect the outcome of Diff-seq is difficult to predict and account for at this stage.

Potential application of Diff-seq to more complex genomes opens several exciting possibilities. For example, carrying out Diff-seq during exome capture may provide improved coverage of relevant polymorphic regions, potentially decreasing the sequencing resources required for comparable amounts of information. Furthermore, variant discovery in circulating tumor cells has been demonstrated through arduous enrichment approaches coupled with massive sequencing efforts involving multiple independent library preparations to distinguish SNPs from errors introduced during amplification and sequencing (42). Approaches for detecting somatic mutations associated with cancer from cell-free DNA likewise involve extremely deep, molecularly tagged sequencing. Diff-seq, coupled with significant improvements in efficiency, has the potential to substantially reduce the required sequencing capacity of these workflows by moving the mismatch detection ‘up front’ and focusing sequencing capacity on polymorphic regions.

## DATA AVAILABILITY

The custom written software used for data analysis is available at https://github.com/Sherlock-Lab/Diff-seq, https://zenodo.org/badge/latestdoi/101428770.

The datasets generated and analyzed during the current study are available in the Sequencing Read Archive, under study accession SRP116147 (https://submit.ncbi.nlm.nih.gov/subs/sra/). Samples description can be found under bioproject accession number PRJNA397463. PCR sequences of population samples can be found under accession numbers GQ212684 and GQ212685 for sample with PID 30269 and GQ206339 and GQ206337 for sample with PID 5248 (8).

## Supplementary Material

Supplementary Data
