# Supplementary material for: Diff-seq: A high throughput sequencing-based mismatch detection assay for DNA variant enrichment and discovery
Source: Nucleic Acids Res. 2018 Jan 19;46(7):e42. doi: 10.1093/nar/gky022 (PMC5909455; doi:10.1093/nar/gky022)
Supplement: Supplementary Data [file gky022_supp.zip › nar-03139-met-g-2017-File006.pdf]

Supplementary figures and tables legends

Figure S1. Read processing flowchart. Libraries were prepared as shown in Figure 1, amplified, quantified and sequenced on an Illumina Miseq. The library structure for 2 reads that map to the forward and reverse strand of the reference is shown on top. Read processing includes 5 steps: merging of paired end reads, read deduplication, adaptor trimming, which includes trimming of the buffer sequence downstream of read 1, read filtering, and aligning to the reference.

Figure S2. Differential sequencing coverage is high at and around polymorphic sites. 2 Diff-seq libraries (control and with a SNP at position 477) derived from a 1 kb sequence were prepared as described in Figure 1 and sequenced on a fraction of a MiSeq run. (**A**) Example of aligned forward and reverse reads around a SNP position, annotated in bold in the reference sequence. The positions within the aligned reads that are considered in Diff-seq coverage are highlighted in black and bold font. (**B**) Total coverage frequencies are plotted against the position on the reference. Forward and reverse reads are plotted with a positive and negative sign, respectively. Zoom-in the x-axis plots around the variant position (and the respective control) are shown below. (**C**) Diff-seq coverage frequencies are plotted against the position on the reference as in B, and color-coded according to nucleotide identity.

Figure S3. Differential sequencing application to a model substrate. Differential sequencing libraries derived from a 1 kb sequence were prepared and plotted as in Figure 2. (**A-C**) Samples with a single variant position (477). (**D** and **E**) Samples with the same variation in 2 different positions. (**F**) Control sample (matched library) is the same shown in Figure 2 with zoom in the x-axis, where mismatches are in the rest of the libraries, for comparison. Heterohybrids in the mismatched libraries were generated by hybridization of 2 molecules, mixed at 1:1. When more than 1 mismatch were assayed (D and E), different hybridization reactions were mixed iso-stoichiometrically.

Figure S4. Allele contributions on the Diff-seq signal of the SNP positions between the viral clones. (A) Mismatch- and allele-specific contributions to the total and strand-specific signals for each of the 59 SNP positions. We assumed that matched molecules in these positions gave no signal at all. Forward and reverse allele-specific biases, as well as their average, total and maximum, and the log2 Diff-seq coverage frequencies are shown. The average and maximum allele biases refer to the forward and reverse allele bias, whereas the total allele bias does not differentiate between strands. The sites have been ordered first by the reference-alternative contributing alleles and then by their trinucleotide context. U: upstream, D: downstream, R: reference, A: alternative. Black boxes correspond to missing values. The positions on the reference are annotated on the left. **(B)** Allele-specific contributions on the signal of each type of variation. The fraction of each contributing allele is plotted for each reference-alternative pair for all alleles in both orientations. The median and upper and lower quartiles are annotated with black vertical lines on the colored boxes. The number of observations for each reference-alternative pair is shown at the bottom.

Figure S5. Positions on the reference ranked based on the values of different predictors for the 5% rare variant frequency dataset. The sum of the per strand enrichment scores for each of the following variables was ranked for all positions of the reference: (**A**) Total Diff-seq coverage,

(**B**) minor allele Diff-seq coverage, (**C**) major allele Diff-seq coverage per major allele identity, (**D**) minor allele Diff-seq coverage per major and minor allele identity, (**E**) major allele Diff-seq coverage within trinucleotide context, (**F**) minor allele Diff-seq coverage within trinucleotide context. SNPs, dense SNPs and no variant positions (match) are annotated. ROC curves for these variables are shown in Figure 3E.

Figure S6. Comparison between Diff-seq and Nextera library preparations for the detection of variation across different variation frequencies. The log2 non-reference allele coverage frequencies for each position of the reference was plotted for the Diff-seq (y-axis) against Nextera (x-axis) library preparations. The positions colored gray, black and cyan represent non-variant positions, SNPs and dense SNPs, respectively, and the rare:frequent variant ratios are as shown.

Table S1. Oligos used in this study.

Table S2. Diff-seq library characteristics of the libraries used in this study.

Figure S1

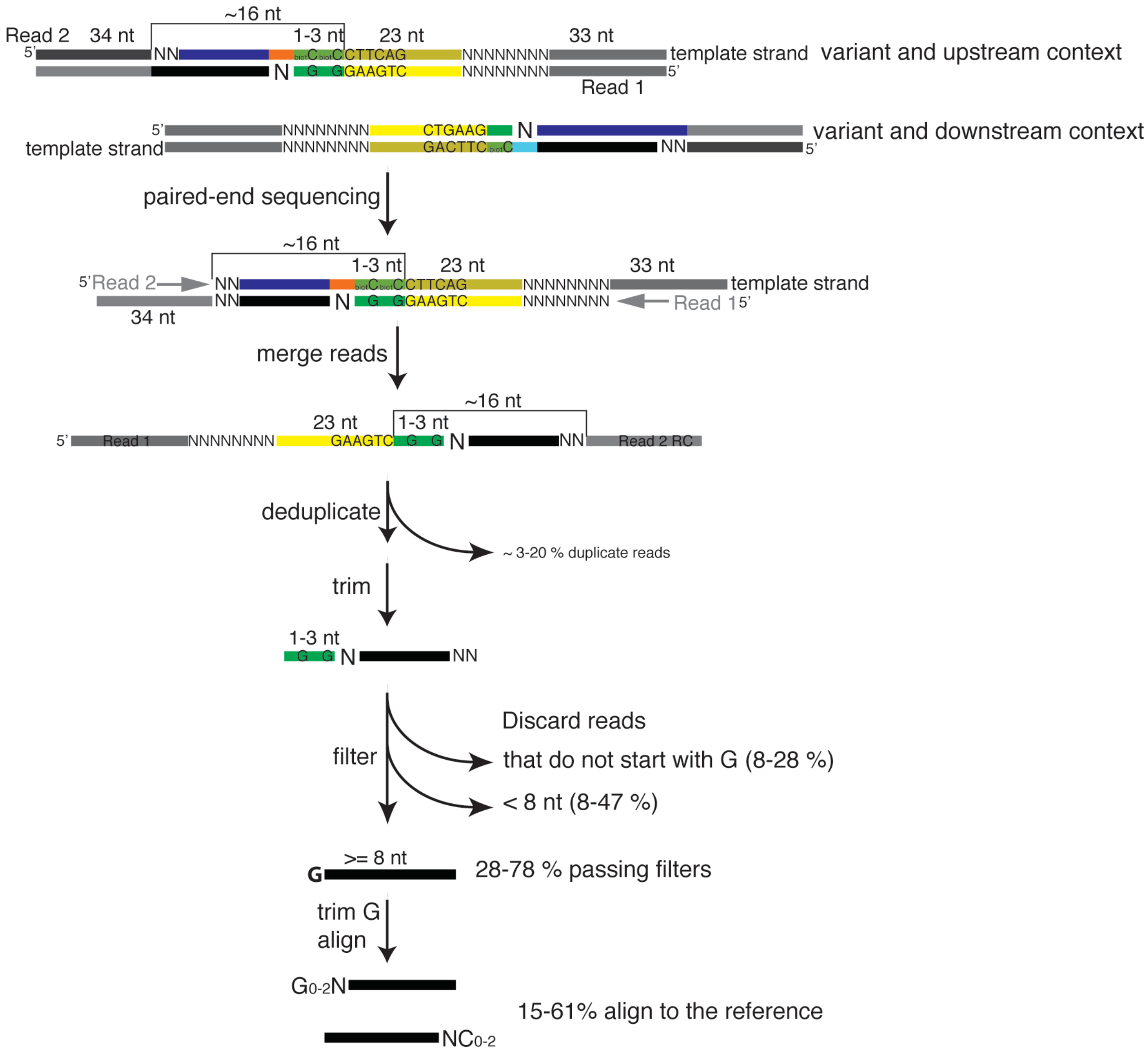
~16 nt
Read 2
34 nt
1-3 nt
23 nt
33 nt
template strand
variant and upstream context
Read 1
variant and downstream context
template strand
paired-end sequencing
5'Read 2
Read 1 5'
merge reads
deduplicate
~ 3-20 % duplicate reads
trim
filter
Discard reads
that do not start with G (8-28 %)
< 8 nt (8-47 %)
>= 8 nt
28-78 % passing filters
trim G
align
15-61% align to the reference

Figure S2

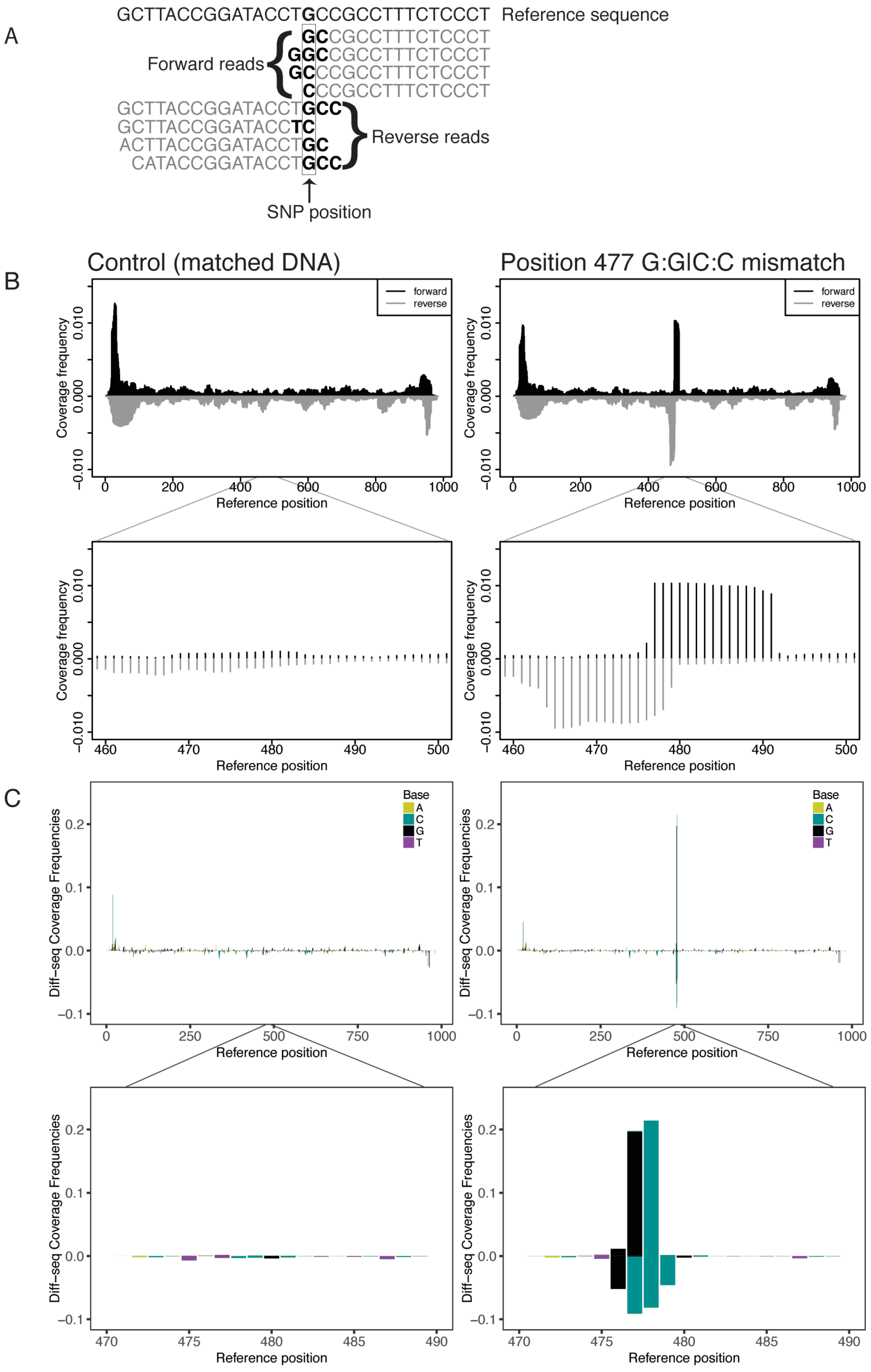

A
GCTTACCGGATACCTGCCGCCTTTCTCCCT Reference sequence
Forward reads
GCCGCCTTTCTCCCT
GGCCGCCTTTCTCCCT
GCCCGCCTTTCTCCCT
CCCGCCTTTCTCCCT
GCTTACCGGATACCTGCC
GCTTACCGGATACCTC
ACTTACCGGATACCTGC
CATACCGGATACCTGCC
Reverse reads
SNP position
B
Control (matched DNA)
Position 477 G:GIC:C mismatch
forward
reverse
Coverage frequency
Reference position
C
Base
A
C
G
T
Diff−seq Coverage Frequencies
Reference position

Figure S3

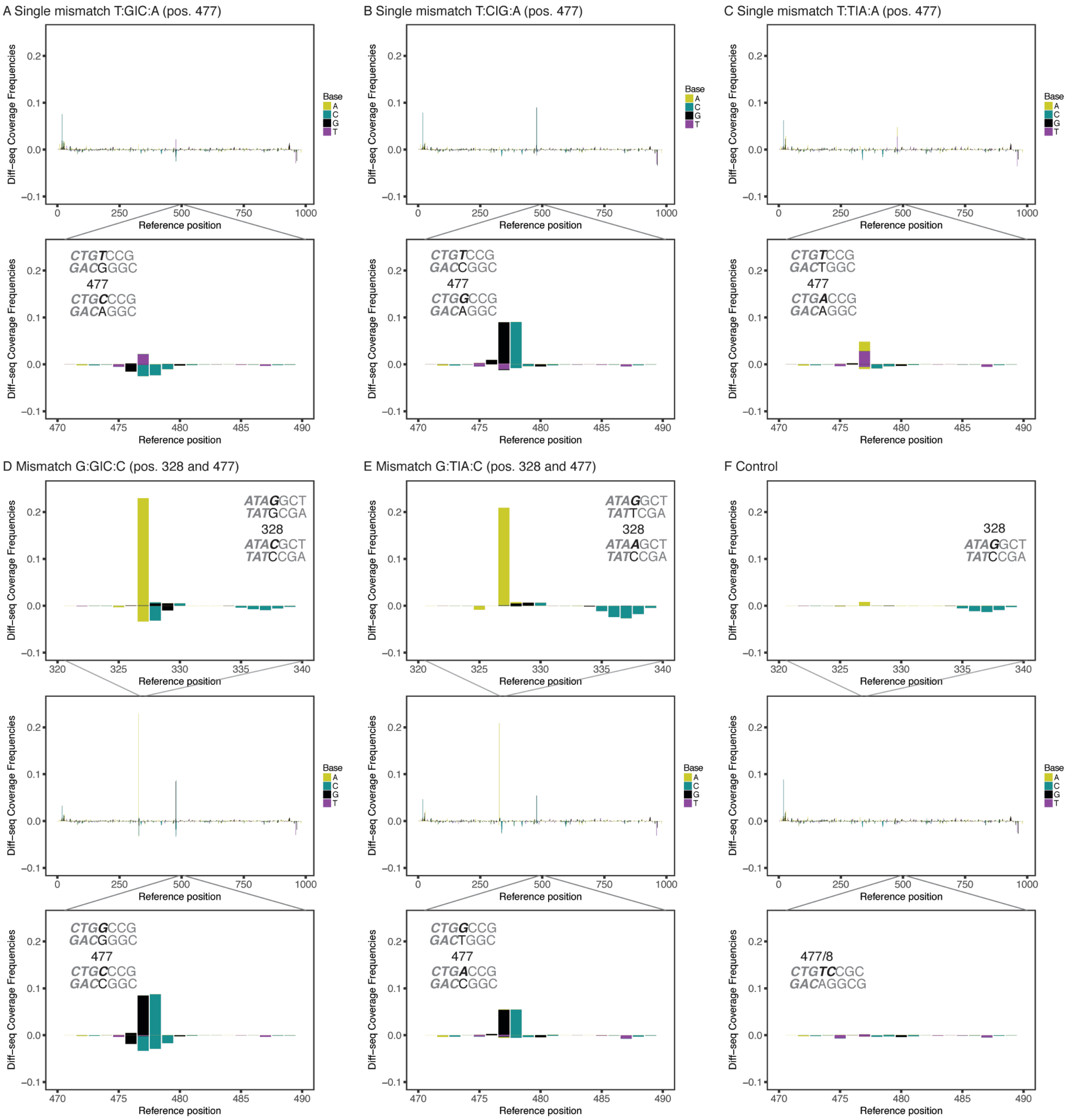
A Single mismatch T:GIC:A (pos. 477)
B Single mismatch T:CIG:A (pos. 477)
C Single mismatch T:TIA:A (pos. 477)
D Mismatch G:GIC:C (pos. 328 and 477)
E Mismatch G:TIA:C (pos. 328 and 477)
F Control
Diff−seq Coverage Frequencies
Reference position
Base
A
C
G
T
CTGTCCG
GACGGGC
477
CTGCCCG
GACAGGC
CTGTCCG
GACCGGC
477
CTGGCCG
GACAGGC
CTGTCCG
GACTGGC
477
CTGACCG
GACAGGC
ATAGGCT
TATGCGA
328
ATACGCT
TATCCGA
ATAGGCT
TATTCGA
328
ATAAGCT
TATCCGA
328
ATAGGCT
TATCCGA
CTGGCCG
GACGGGC
477
CTGCCCG
GACCGGC
CTGGCCG
GACTGGC
477
CTGACCG
GACCGGC
477/8
CTGTCCGC
GACAGGCG

Figure S4

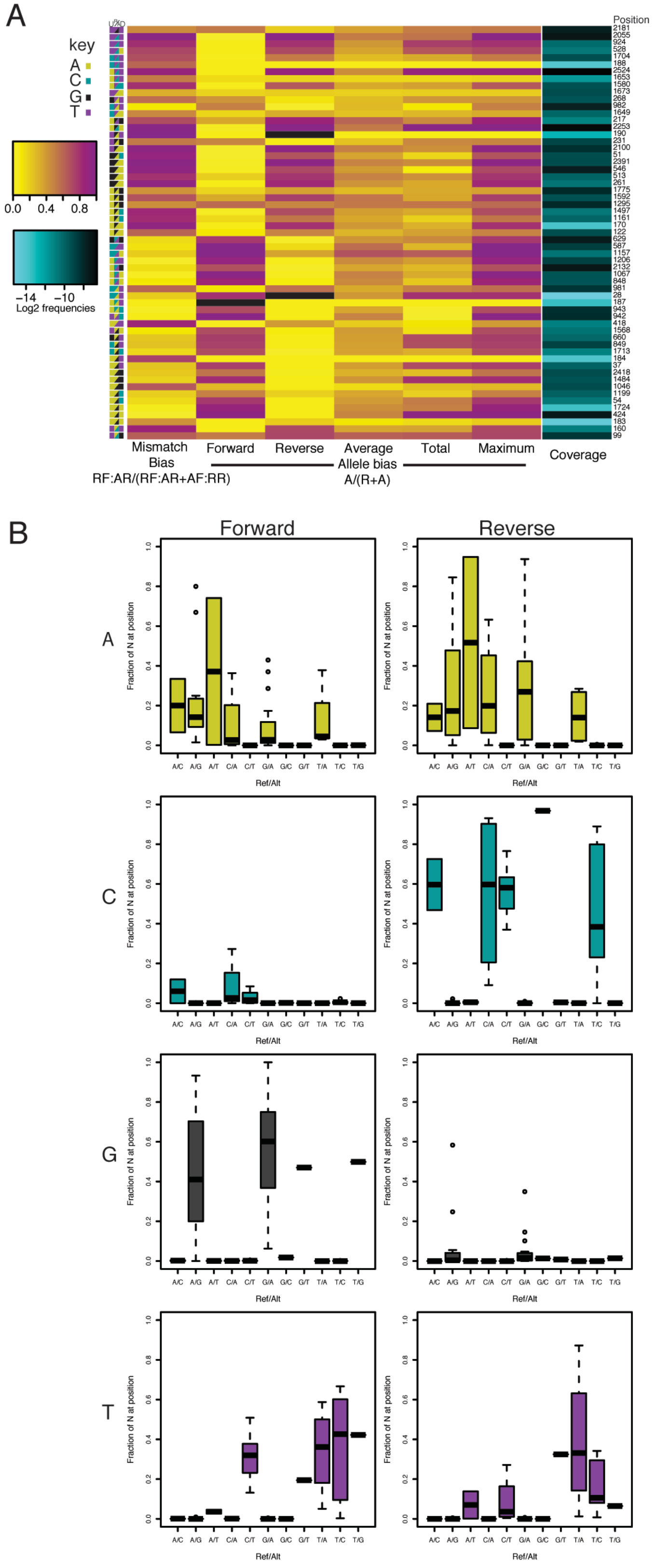

Ref/Alt	A/C	A/G	A/T	C/A	C/G	C/T	G/A	G/C	G/T	T/A	T/C	T/G
Number of observations	2	14	2	4	0	7	15	1	1	4	8	1

Figure S5

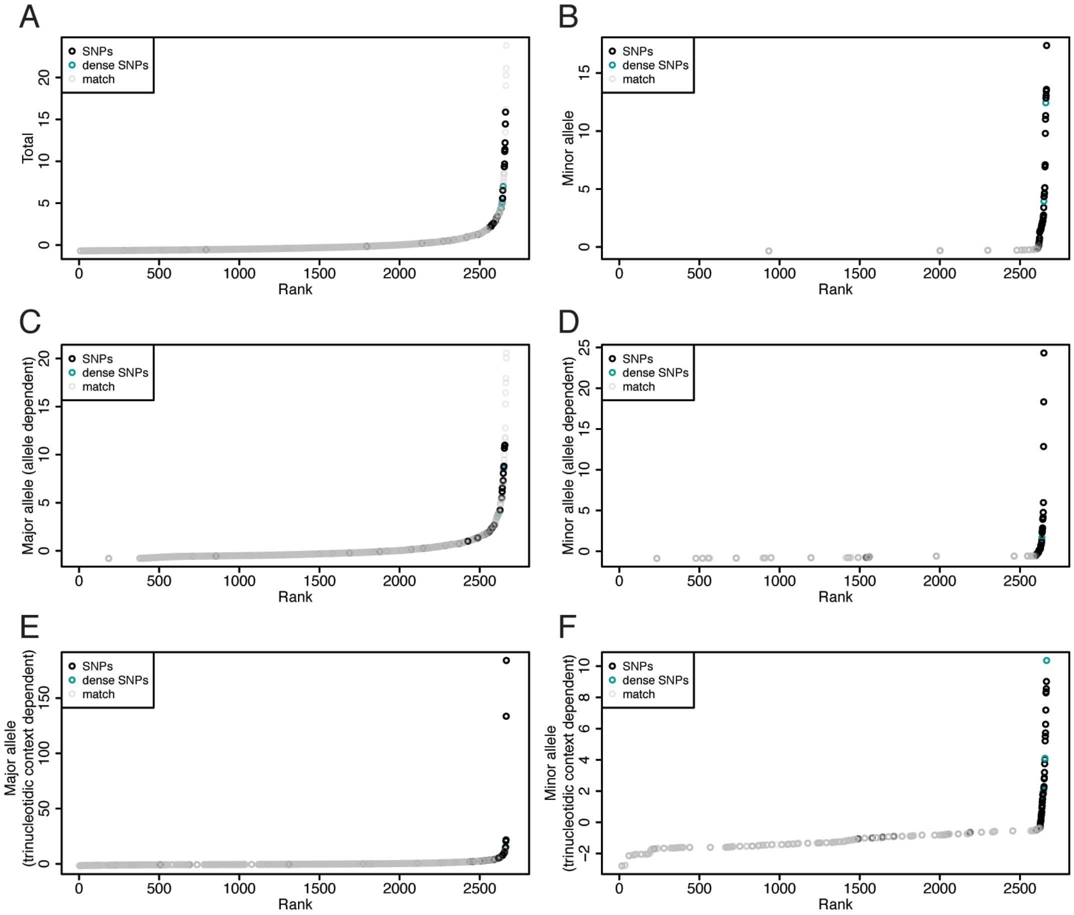
A
B
C
D
E
F
SNPs
dense SNPs
match
Total
Minor allele
Major allele (allele dependent)
Minor allele (allele dependent)
Major allele
(trinucleotidic context dependent)
Minor allele
(trinucleotidic context dependent)
Rank
0
500
1000
1500
2000
2500

Figure S6

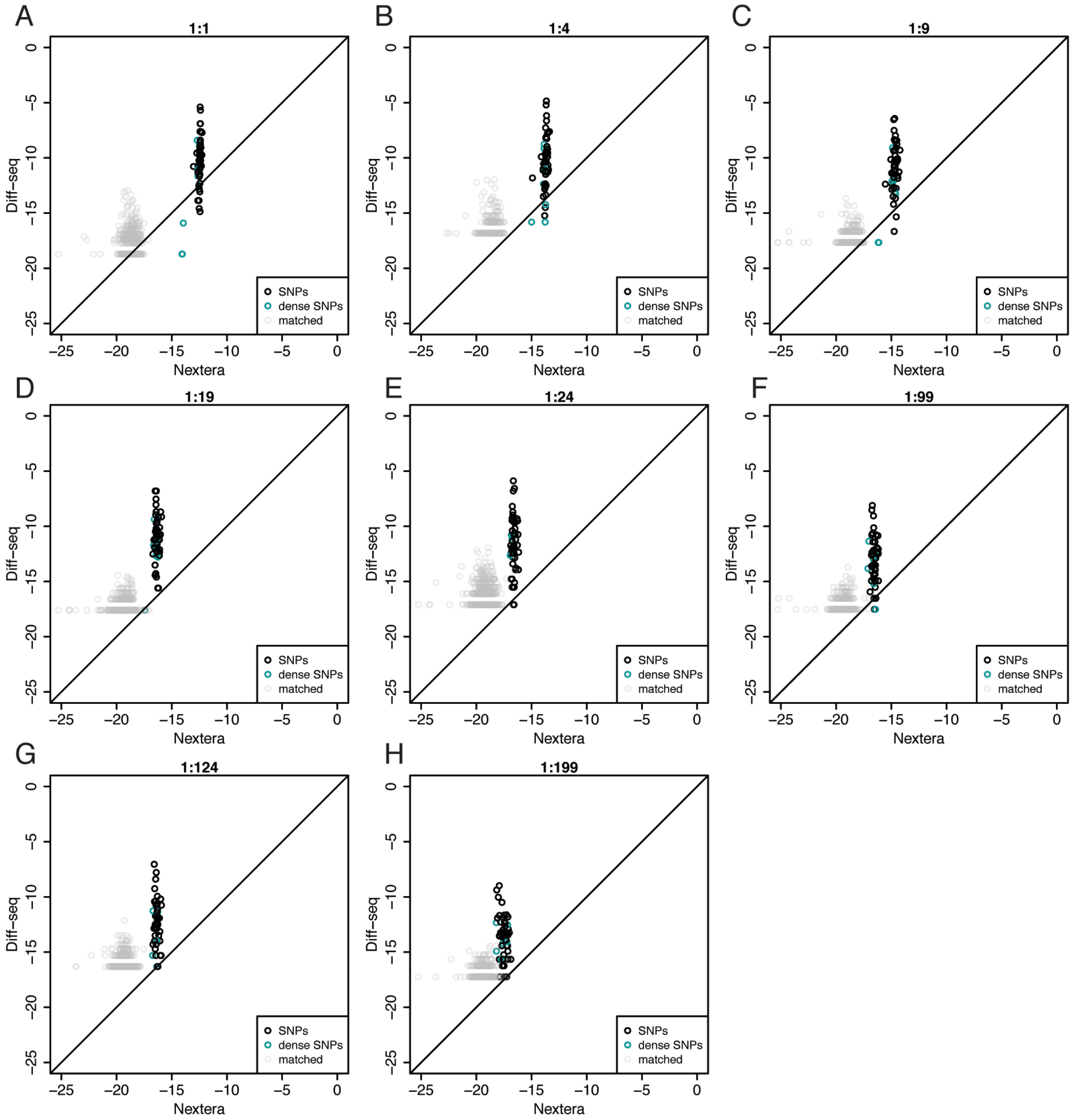
A
1:1
B
1:4
C
1:9
D
1:19
E
1:24
F
1:99
G
1:124
H
1:199
Diff−seq
Nextera
SNPs
dense SNPs
matched

Table S1

Oligo	Sequence	Usage
VK1	5’-CTTACCGGATACCTGCCCGCCTTTCTCCCTTC-3'	QuickChange PCR
VK2	5’-CTTACCGGATACCTGGCCGCCTTTCTCCCTTC-3'	QuickChange PCR
VK3	5’-CTTACCGGATACCTGACCGCCTTTCTCCCTTC-3'	QuickChange PCR
VK5	5’-CTTACCGGATACCTGTACGCCTTTCTCCCTTC-3'	QuickChange PCR
VK7	5’-GAAGGGAGAAAGGCGGGCAGGTATCCGGTAAG-3'	QuickChange PCR
VK8	5’-GAAGGGAGAAAGGCGGCCAGGTATCCGGTAAG-3'	QuickChange PCR
VK9	5’-GAAGGGAGAAAGGCGGTCAGGTATCCGGTAAG-3'	QuickChange PCR
VK11	5’-GAAGGGAGAAAGGCGTACAGGTATCCGGTAAG-3'	QuickChange PCR
VK19	5’-GCTGGCGTTTTTCCATAAGCTCCGCCCCCCTGAC-3'	QuickChange PCR
VK21	5’-GCTGGCGTTTTTCCATACGCTCCGCCCCCCTGAC-3'	QuickChange PCR
VK22	5’-GTCAGGGGGGCGGAGCTTATGGAAAAACGCCAGC-3'	QuickChange PCR
VK24	5’-GTCAGGGGGGCGGAGCGTATGGAAAAACGCCAGC-3'	QuickChange PCR
VK41	5'-CATGACCCAGTCACGTAGCGATAG-3'	pET17b variants amplification
VK42	5'-CTCATGACCAAAATCCCTTAACGTGAG-3'	pET17b variants amplification
DAo43	5’-CAGAGCCAACAGCCCCACCAG-3’	Viral clones amplification
DAo44	5’-TGCTGTCCCTGTAATAAACCCGAAAATTTTGAATTTTTG-3’	Viral clones amplification
DAo83	5’-GAGCATGAGCGCTCGTCT*CTGAAG*GN-3’	1st ligation reaction
DAo84	5’-GAGCATGAGCGCTCGTCT*CTGAAG*GGN-3’	1st ligation reaction
DAo85	5’-GAGCATGAGCGCTCGTCT*CTGAAG*GGGN-3’	1st ligation reaction
DAo97	5’(Phos)-*CTTCAG*AGACGAGCGCTCATGCTNNNNNNNNCTGTCTCTTATACACATCTGACGCTGCCGACGA-3’(Phos)	1st ligation reaction
DAo98	5’-GTCTCGTGGGCTCGGAGATGTGTATAAGAGACAGNN-3’	2nd ligation reaction
DAo99	5’(Phos)-CTGTCTCTTATACACATCTCCGAGCCCACGAGAC-3’(Phos)	2nd ligation reaction
DAo100	5’-CAAGCAGAAGACGGCATACGAGATCTAGTACGGTCTCGTGGGCTCGGAGATGTGTATAAGAGACAG-3’	Library 1st PCR reaction
DAo101	5’-CAAGCAGAAGACGGCATACGAGATGCTCAGGA GTCTCGTGGGCTCGGAGATGTGTATAAGAGACAG-3’	Library 1st PCR reaction
DAo102	5’-AATGATACGGCGACCACCGAGATCTACACAGAGTAGA TCGTCGGCAGCGTCAGATGTGTATAAGAGACAG-3’	Library 1st PCR reaction
DAo103	5’-AATGATACGGCGACCACCGAGATCTACACGTAAGGAG TCGTCGGCAGCGTCAGATGTGTATAAGAGACAG -3’	Library 1st PCR reaction
DAo106	5’-CAAGCAGAAGACGGCATACGAGATTTCTGCCTGTCTCGTGGGCTCGGAGATGTGTATAAGAGACAG-3’	Library 1st PCR reaction
DAo107	5’-CAAGCAGAAGACGGCATACGAGATTCGCCTTA GTCTCGTGGGCTCGGAGATGTGTATAAGAGACAG-3’	Library 1st PCR reaction
DAo108	5’-AATGATACGGCGACCACCGAGATCTACACTATCCTCTTCGTCGGCAGCGTCAGATGTGTATAAGAGACAG-3’	Library 1st PCR reaction
DAo109	5'-AATGATACGGCGACCACCGAGATCTACACACTGCATATCGTCGGCAGCGTCAGATGTGTATAAGAGACAG-3'	Library 1st PCR reaction
DAo117	5’-AATGATACGGCGACCACCGAGATCTACACCTCTCTATTCGTCGGCAGCGTCAGATGTGTATAAGAGACAG-3’	Library 1st PCR reaction
DAo-AdC	5’-AATGATACGGCGACCACCGAGATCTACAC-3’	Library qPCR and 2nd PCR reaction
DAo-AdD	5’-CAAGCAGAAGACGGCATACGAGAT-3’	Library qPCR and 2nd PCR reaction
